# Proteomic Characterization of Colorectal Cancer Cells versus Normal-Derived Colon Mucosa Cells: Approaching Identification of Novel Diagnostic Protein Biomarkers in Colorectal Cancer

**DOI:** 10.3390/ijms21103466

**Published:** 2020-05-14

**Authors:** Maja Ludvigsen, Louise Thorlacius-Ussing, Henrik Vorum, Mary Pat Moyer, Mogens Tornby Stender, Ole Thorlacius-Ussing, Bent Honoré

**Affiliations:** 1 Department of Clinical Medicine, Aarhus University Hospital, Aarhus, DK-8200 Aarhus N, Denmark; majlud@rm.dk; 2 Department of Hematology, Aarhus University Hospital, Aarhus, DK-8200 Aarhus N, Denmark; 3 Department of Biomedicine, Aarhus University, Aarhus, DK-8000 Aarhus C, Denmark; louise.thorlacius-ussing@regionh.dk; 4 Department of Gastrointestinal Surgery, Aalborg University Hospital, Aalborg, DK-9000 Aalborg, Denmark; mogens.stender@rn.dk (M.T.S.); otu@rn.dk (O.T.-U.); 5 Department of Clinical Medicine, Aalborg University, Aalborg, DK-9000 Aalborg, Denmark; henrik.vorum@rn.dk; 6 Department of Ophthalmology, Aalborg University Hospital, Aalborg, DK-9000 Aalborg, Denmark; 7 INCELL Corporation, LLC, San Antonio, TX 78249, USA; mpmoyer@incell.com

**Keywords:** colorectal adenocarcinoma, colorectal cancer, protein expression, proteomics, biomarkers, cellular model system

## Abstract

In the western world, colorectal cancer (CRC) is the third most common cause of cancer-related deaths. Survival is closely related to the stage of cancer at diagnosis striking the clinical need for biomarkers capable of early detection. To search for possible biological parameters for early diagnosis of CRC we evaluated protein expression for three CREC (acronym: *C*ab45, *r*eticulocalbin, *E*RC-55, *c*alumenin) proteins: reticulocalbin, calumenin, and ERC-55 in a cellular model consisting of a normal derived colon mucosa cell line, NCM460, and a primary adenocarcinoma cell line of the colon, SW480. Furthermore, this cellular model was analyzed by a top-down proteomic approach, 2-dimensional polyacrylamide gel electrophoresis (2D-PAGE) and liquid chromatography–tandem mass spectrometry (LC–MS/MS) for novel putative diagnostic markers by identification of differentially expressed proteins between the two cell lines. A different colorectal carcinoma cell line, HCT 116, was used in a bottom-up proteomic approach with label-free quantification (LFQ) LC–MS/MS. The two cellular models gave sets of putative diagnostic CRC biomarkers. Various of these novel putative markers were verified with increased expression in CRC patient neoplastic tissue compared to the expression in a non-involved part of the colon, including reticulocalbin, calumenin, S100A6 and protein SET. Characterization of these novel identified biological features for CRC patients may have diagnostic potential and therapeutic relevance in this malignancy characterized by a still unmet clinical need.

## 1. Introduction

Colorectal cancer (CRC) is among the leading causes of death in the Western countries and considerable effort are spent identifying diagnostic biomarkers. Mean overall survival is closely related to the stage at time of diagnosis. The 5-year overall survival rates are around 50% [1,2,3]. In the western world, CRC is the third most common cause of cancer deaths, striking the clinical need for biomarkers able to identify the malignancy at an early stage of cancer [4].

Molecular genetic analyses have revealed numerous mutations underlying the pathogenesis of CRC. However, most identified mutations seem only to be present in subsets of the patients and only well-known suppressor genes, e.g., p53, APC, and KRAS are mutated in the majority of patients [5]. Several factors may influence the proteomic machinery and affect the final properties of the encoded protein at several levels: translational inhibition/activation, posttranslational modifications, inhibition/activation of the proteins, specific degradations, etc. Consequently, there can be considerable discrepancy between the expression level of RNA and that of the corresponding protein product [6]. Proteins are the molecules that execute most cellular functions and many regulatory processes take place at the protein level. Furthermore, the majority of all current therapies are directed at proteins. This prompted us to analyze putative CRC diagnostic markers at the protein level. 

In recent years, extensive research has intended to identify biomarkers for CRC in blood and stool but in spite of this, these markers are still of limited clinical value. Furthermore, large population-based programs have been established to screen for CRC [7]. The screening programs are primarily based on fecal test for blood. However, in Western cultures, handling has reduced the compliance to programs. In contrast, the collection of a blood sample is usually more acceptable for the general population. Consequently, search for both genetic and non-genetic markers has increased during the last decades. The only biomarker so far used in clinical practice is carcinoembryonic antigen (CEA), but its value as an isolated screening and prognostic marker is limited and it is currently recommended as a supplement approach in monitoring CRC recurrence [8,9,10,11,12].

Protein expression analysis of tissue may be difficult due to the heterogeneous nature with the presence of a number of different cell types that each may vary in protein expression. A cellular model system is inherently more homogeneous due to the restricted number of cell types present, although even a cell type may show some degree of heterogeneity and be sensitive to the conditions of cultivation. In the present study, we have compared the protein expression of a normal derived colon mucosa cell line, NCM460 [13] with the expression in two cell lines, one cell line established from a primary adenocarcinoma of the colon, SW480 [14], and another cell line established from a human colonic carcinoma, HCT 116 [15]. These model cell culture systems possess a number of differences [16] and have previously been analyzed for transcript expression [17]. Amongst seven differentially expressed transcripts, Nimmrich et al. found the transcript of the CREC member reticulocalbin to be upregulated in the cancer cell line SW480 as compared with the normal-derived cell line, NCM460 [17]. Several reports have revealed differential expression of the CREC proteins by studying diseases or model systems such as cancer cells, or cells exposed to various forms of treatment or “stress” and comparing these with normal conditions [18,19]. We investigated the protein expression of three CREC proteins which previously have been related to the development of cancer. Reticulocalbin has been shown to be implicated in breast, non-small cell lung, and hepatic cancer [20,21,22]. The transcript expression of calumenin has been shown to be correlated to risk-groups in mesothelioma and down-regulation has been associated with lung squamous cell carcinomas [23,24]. ERC-55 expression has been shown in correlation to tumor-mass in breast cancer and in more aggressive forms of cervical cancer together with a study showing downregulation of ERC-55 in regard to prostate cancer [25,26,27]. We investigated the potential of reticulocalbin, calumenin and ERC-55 as diagnostic markers, initially in the SW480 cellular model and then for two of them in confirmatory analyses with CRC patient tissue samples from: (i) the central part of the tumor and (ii) the peripheral part of the tumor as well as (iii) a tumor free part of the colon.

Furthermore, we identify new putative protein markers for diagnostic use in CRC by proteomic comparison of the expression levels in two different cellular models and found numerous differentially expressed proteins. The expression patterns of selected identified proteins, i.e., S100A4, S100A6, retinol-binding protein I (RBP), Protein SET, and endoplasmin (HSP90B1) were further evaluated in the cell lines and/or in CRC patient tissue by western blotting.

## 2. Results

### 2.1. CREC Protein Expression in NCM460 and SW480 Cell Lines and in Patient Colorectal Cancer (CRC) Tissue

**Figure 1:**
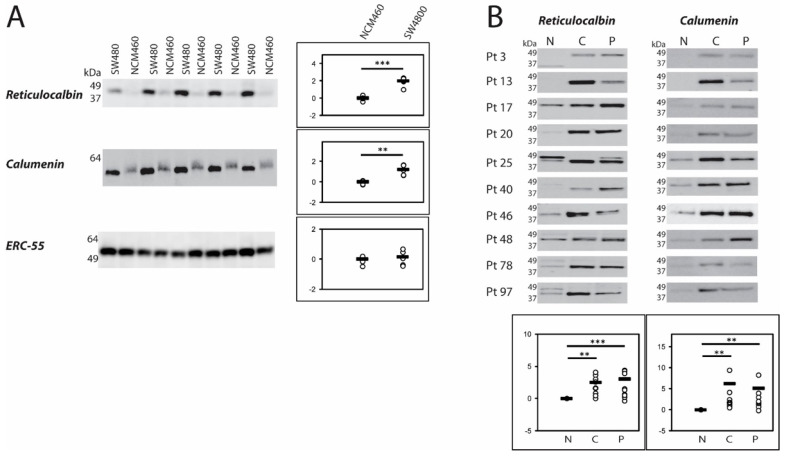
Western blotting of three CREC family members. (**A**) NCM460 and SW480 cells were grown in separate rounds and harvested prior to total protein concentration determination and equal amounts of protein were loaded on the gels. Pixel intensity was measured in each band and the mean value of NCM460 was used as a reference set to 1. *p* values were calculated with Mann-Whitney U-test. Relative pixel intensities are depicted at a log_2_-transformed scale in the dot plots. (**B**) Colorectal cancer (CRC) patient tissue was collected during bowel resection for CRC. Proteins were solubilized by homogenization in lysis buffer prior to total protein concentration determination and equal amounts of protein were loaded on the gels. Pixel intensity was measured in each band and the value of each non-involved (N) band were set to 1 and the peripheral (P) and central (C) samples were calculated relative to the non-involved tissue value in each patient. *p* values were calculated with Wilcoxon signed-rank test. Relative pixel intensities are depicted at a log_2_-transformed scale in the dot plots. ** *p* < 0.01; *** *p* < 0.001.

We have compared the protein expression of three members of the CREC family reticulocalbin, calumenin and ERC-55 in a CRC cellular model, i.e., a normal derived colon mucosa (NCM460) cell line and a cell line established from a primary adenocarcinoma of the colon (SW480) [13,14]. Reticulocalbin and calumenin are both highly increased at the protein level in the cancer cell line (*p* < 0.001, *p* < 0.01) (Figure 1A). Moreover, the protein band recognized by the calumenin antibody decreases slightly in size in the cancer cell line as compared with the normal derived cell line. The third CREC member, ERC-55, is expressed at similar levels in the normal derived cell line, NCM460, as in the cancer cell line, SW480.

Subsequently, we analyzed the protein expression of reticulocalbin and calumenin in biopsies from 10 patients diagnosed with CRC (Table 1). Since ERC-55 was present at similar levels in the two cell lines we saved the patient biopsies for other putative markers. From each patient a sample was taken from the central part of the tumor (C), the peripheral part of the tumor (P) as well as from a non-involved part of the colon (N). Reticulocalbin is highly expressed in the central part as well as in the peripheral part of the tumor (*p* < 0.01, *p* < 0.001) compared to the expression of the non-involved part of the colon. A similar pattern is observed for calumenin (*p* < 0.01, *p* < 0.01) (Figure 1B).

### 2.2. Top-Down Proteomic Comparison of NCM460 and SW480 Cell Lines

**Figure 2:**
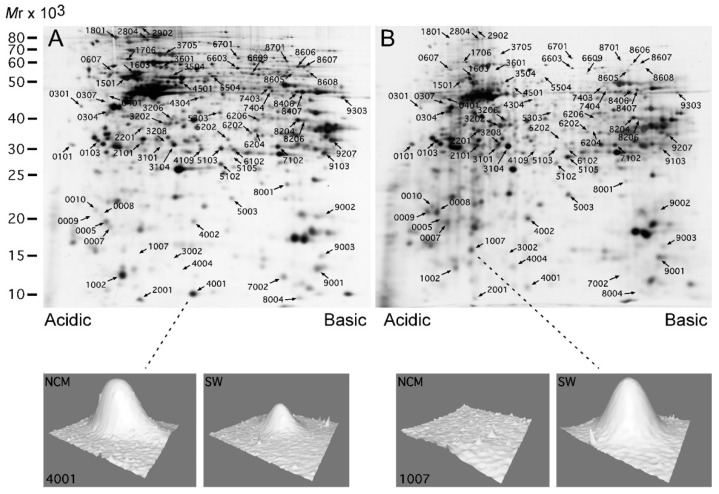
Proteomic analysis of NCM460 and SW480 using a top-down proteomic strategy. A representative gel from each group (**A**) NCM460 and (**B**) SW480, molecular mass is given in kDa. NCM460 and SW480 cells were grown in separate rounds and harvested prior to total protein concentration determination and equal amounts of protein were loaded on the gels. Each group consisted of 6 samples. Comparative analyses of the spots were done with the PDQuest software. All differentially identified spots with 2-fold or more increased/decreased expression and with significance level (*p* < 0.05) with Mann-Whitney U-test are shown on the representative gels in the figure.

We further analyzed the NCM460/SW480 cellular model system by performing a proteomic comparison using 2-dimensional polyacrylamide gel electrophoresis (2D-PAGE) in order to detect protein spots that were significantly and at least 2-fold differentially expressed between the two cell lines. This analysis revealed 73 spots changing in expression. All spots were excised for protein identification. Twenty-five spots (16 with protein identifications) were upregulated and 48 spots (22 with protein identifications) were downregulated (Figure 2) in the colon cancer cell line (SW480) compared to the protein expression of the normal-derived colon cell line (NCM460). In total 38 spots were identified with at least one peptide by liquid chromatography–tandem mass spectrometry (LC–MS/MS). Details are given in Table 2.

### 2.3. Bottom-up Proteomic Comparison of NCM460 and HCT 116 Cell Lines

**Figure 3:**
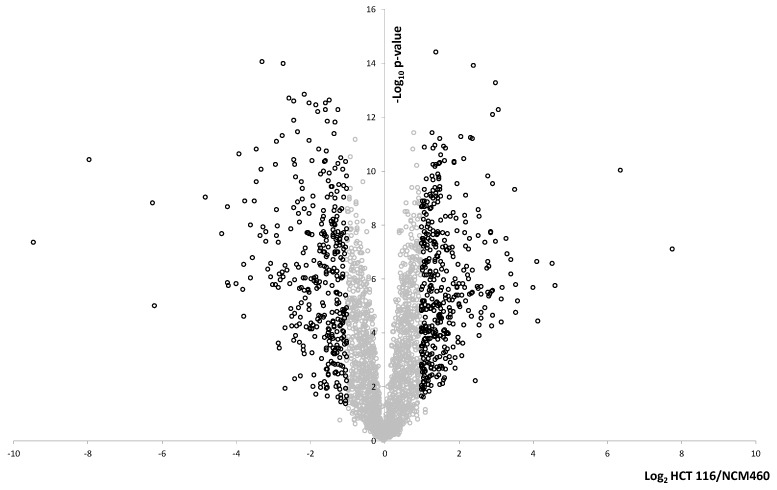
Proteomic analysis of NCM460 and HCT 116 using a bottom-up proteomic strategy. Proteins were analyzed by label-free quantification (LFQ) liquid chromatography–tandem mass spectrometry (LC–MS/MS). MaxQuant and Perseus were used for data analysis. The log_2_ transformed relative expression levels of HCT 116 versus NCM460 are plotted on the x-axis and on the y-axis are plotted the log_10_ transformed *p*-values. Unchanged proteins are indicated with grey circles. 901 proteins were significantly (*p* < 0.05) at least 2-fold differentially expressed. 777 of these are indicated with black circles and 57 were detected in all HCT 116 samples and were below detection limit in all NCM460 samples while 67 proteins were detected in all NCM460 and were below detection limit in all HCT 116 samples. They could not be pictured in the volcano plot, but are included in supplementary Table S1.

The NCM460/HCT 116 cellular model system was analyzed by label-free quantification (LFQ) LC–MS/MS proteomics in order to detect proteins that were significantly and at least 2-fold differentially expressed in this cellular model system. We found 901 proteins to be differentially expressed, 465 upregulated and 436 downregulated, Figure 3. The differentially expressed proteins are listed in supplementary Table S1. Retinol-binding protein I was highly upregulated in HCT 116 and was below detection limit in NCM460 in line with the 2D-PAGE results (Figure 2, spot 1007). None of the CREC proteins, reticulocalbin, calumenin or ERC-55 were changed in this system. Protein SET and endoplasmin were not changed either. Only one of the S100 proteins was detected, S100A10, and it was not changed.

### 2.4. Immunologic Evaluation of Protein Expression

**Figure 4:**
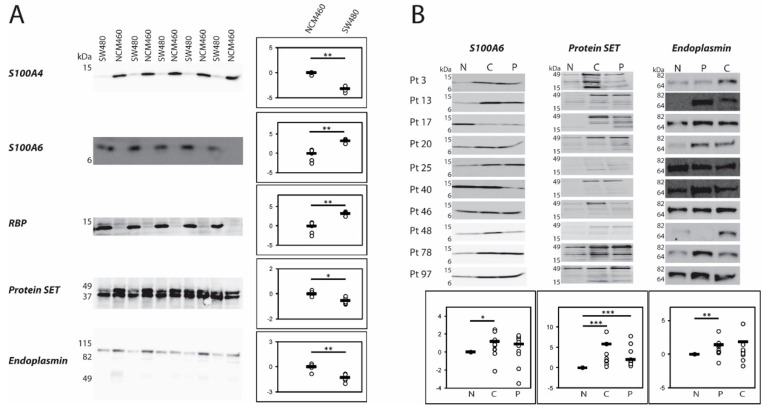
Western blotting of potential markers. (**A**) NCM460 and SW480 cells were grown in separate rounds and harvest prior to total protein concentration determination and equal amount of protein were loaded on the gels. Pixel intensity was measured in each band and the mean value of NCM460 was used as reference set to 1. Total pixel intensity from the two bands for protein SET showed no significant difference (*p* = 0.2). However, quantifying the top band alone showed significant up-regulation in NCM460 (*p* < 0.05) whereas the lower band showed no significant difference (*p* = 0.9). *p* values were calculated with Mann-Whitney U-test. Relative pixel intensities are depicted at a log_2_-transformed scale in the dot plots. (**B**) CRC patient tissue was exercised from CRC during resection for CRC. Proteins were solubilized by homogenization in lysis buffer prior to total protein concentration determination and equal amounts of protein were loaded on the gels. Pixel intensity was measured in each band and the value of each non-involved (N) band was set to 1 and the periphery (P) and central (C) samples were calculated relative to the non-involved value in each patient. *p* values were calculated with Wilcoxon signed-rank test. Quantification of protein SET was done on the total expression (all bands together), the top band alone and the band just below the top band alone which all showed the *p*-values as stated in the figure. To save space, the western blot of protein SET has been compressed in the y-direction as inferred by the range of the molecular masses stated by the marker. Few bands were recognized by the endoplasmin antibody migrating approximately at 40 and 25 kDa (not shown). Relative pixel intensities are depicted at a log_2_-transformed scale in the dot plots. * *p* < 0.05; ** *p* < 0.01; *** *p* < 0.001.

The expression patterns of a number of the identified differentially expressed proteins obtained by 2D-PAGE and by LFQ LC–MS/MS were further evaluated by 1D western blotting of the cell lines, NCM460 and SW480, as well as of tissue from CRC patients (Figure 4).

### 2.5. S100 Proteins

S100A4 (spot 4001) was strongly downregulated in the 2D-PAGE proteomic analysis (Figure 2) and this was confirmed by the western blot analysis which also showed strong downregulation of the protein in the SW480 cancer cell line (Figure 4A). This antibody did not show specific reaction in the patient tissue samples. However, an antibody against another member of the S100 family, S100A6, showed reasonable specificity in both systems. S100A6 is upregulated in the SW480 cancer cell line (*p* < 0.01). In the tissue analysis, we found the protein to be upregulated in the central part of the tumor compared to the non-involved part of the colon (*p* < 0.05) (Figure 4A,B). Only one S100 protein, S100A10, was detected with LFQ LC–MS/MS and it was unchanged in HCT 116.

### 2.6. Retinol-Binding Protein I

The antibody against retinol-binding protein I (Figure 2, spot 1007) reacted strongly with the cancer cell line revealing strong upregulation compared with the normal derived colon cell line (*p* < 0.01) in agreement with the 2D-PAGE proteomic analysis (Figure 2, Figure 4A and Table 2). Technically, it was not possible to analyze the tumor tissue due to unspecific reactions of the antibody (not shown). The LFQ LC–MS/MS proteomic analysis also revealed strong upregulation of this protein in the HCT 116 cell line, supplementary Table S1.

### 2.7. Protein SET

Protein SET (Figure 2, spot 0304) was increased in the 2D-PAGE proteomic analysis of SW480. 1D western blotting of the cell lines revealed two closely migrating bands. The upper band analyzed alone showed significant decrease in the SW480 cell line (*p* < 0.05) while the lower band did not show any significant difference (Figure 4A). When the expression pattern of the protein was analyzed in tumor tissue an even more complex pattern was seen since the antibody reacted with several bands on the western blot suggesting that the protein is fragmented in the non-involved part of colon as well as in the tumor tissue (Figure 4B). We found that the central part as well as the peripheral part of the tumor contained significantly more protein/fragments than the non-involved colon (*p* < 0.001, *p* < 0.001). The LFQ LC–MS/MS proteomic analysis revealed an unchanged level in the HCT 116 cell line.

### 2.8. Endoplasmin

Endoplasmin (Figure 2, spot 1801) with a molecular mass of approximately 80 kDa showed a significant decrease in the cancerous SW480 cell line in the 2D-PAGE proteomic analysis and the 1D western blot of the cell lines confirmed the result (*p* < 0.01) (Figure 4A). The 2D-PAGE proteomic analysis also revealed a fragment of endonuclein (Figure 2, spot 0607) with a molecular mass of approximately 55 kDa to be increased in the cancerous cell line. Analysis of the tumor tissue showed a major band of endoplasmin with a molecular mass of approximately 64 kDa, which was significantly increased in the peripheral part (*p* < 0.01) of the tumor but not in the central part compared to the non-involved part of the colon. Additional minor bands with molecular masses around 40 kDa and 25 kDa were observed in the tissue western blot analyses (not shown). The LFQ LC–MS/MS proteomic analysis revealed an unchanged level in the HCT 116 cell line.

### 2.9. Bioinformatic Enrichment Analysis of SW480 versus NCM460

**Figure 5:**
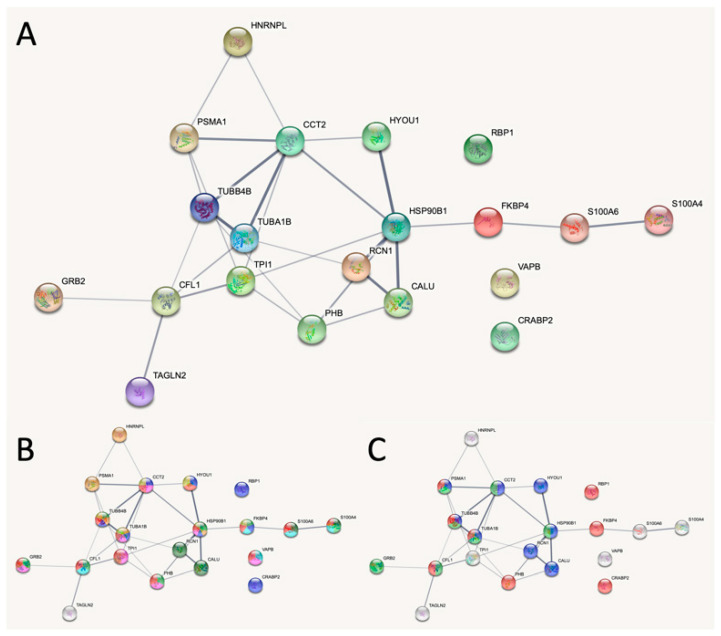
Bioinformatic analysis of differentially expressed proteins. (**A**) STRING v11.0 was used to analyze the genes listed in Table 2 identified with two peptides together with S100A6, calumenin, and reticulocalbin: TAGLN2 (TAGL2_HUMAN), S100A4 (S10A4_HUMAN), CALU (CALU_HUMAN), RCN1 (RCN1_HUMAN), RBP1 (RET1_HUMAN), TPI1 (TPIS_HUMAN), CFL1 (COF1_HUMAN), CRABP2 (RABP2_HUMAN), VAPB (VAPB_HUMAN), HSP90B1 (ENPL_HUMAN), PHB (PHB_HUMAN), PSMA1 (PSA1_HUMAN), CCT2 (TCPB_HUMAN), TUBA1B (TBA1B_HUMAN), GRB2 (GRB2_HUMAN), TUBB4B (TBB4B_HUMAN—updated from TBB2C_HUMAN), HNRNPL (HNRPL_HUMAN), FKBP4 (FKBP4_HUMAN), HYOU1 (HYOU1_HUMAN), S100A4 (S10A4_HUMAN). The 20 proteins have 29 interactions which are significantly more than the expected 6 (*p* < 1.84 × 10^−11^). The analysis indicated that the proteins are at least partially biologically related. (**B**) The 19 proteins involved in various binding functions are indicated with color. Red (13 proteins): ‘protein binding’; blue (8 proteins): ‘small molecule binding’; light green (7 proteins): ‘signaling receptor binding’; pink (7 proteins): ‘enzyme binding’; yellow (6 proteins): ‘purine ribonucleotide binding’; green (5 proteins): ‘calcium ion binding’; brown (5 proteins): ‘RNA binding’; light blue (5 proteins): ‘cytoskeletal protein binding’. (**C**) Proteins identified to be part of Reactome pathways. Red (8 proteins): ‘signal transduction’; blue (8 proteins): ‘metabolism of proteins’; green (7 proteins): ‘immune system’.

The combined set of perturbed proteins from 2D-PAGE and western blot analysis was used for bioinformatic analysis using STRING [28] as given in Figure 5. In order to focus on protein identifications with high reliability we restricted the analysis to proteins identified with at least two peptides. The perturbed proteins have significantly more interactions than expected (*p* < 1.84 × 10^−11^). Thus, the analysis indicated that the proteins are at least partially biologically related. All the 20 proteins belong to the ‘cytoplasmic part’ of the Gene Ontology (GO) classification Cellular Component. With respect to Molecular Function 19 belong to various types of ‘binding’ such as protein binding, small molecule binding, signaling receptor binding, enzyme binding, purine ribonucleotide binding, calcium ion binding, RNA binding and cytoskeletal binding as indicated in Figure 5B. Figure 5C reveals the proteins found to be enriched in Reactome pathways. Eight were found to be part of ‘signal transduction’, eight of ‘metabolism of proteins’, and seven were part of the ‘immune system’. Thus, proteins found to be perturbed in SW480 were mainly cytoplasmic proteins with various binding functions that participate in pathways of signal transduction, protein metabolism, and the immune system.

### 2.10. Bioinformatic Enrichment Analysis of HCT 116 versus NCM460

The differentially expressed proteins by LFQ LC–MS/MS were analyzed by STRING [28]. The proteins had significantly more interactions (7610 edges) than the expected 5050 (*p* < 1 × 10^−16^), see supplementary Figure S1. The large majority of proteins, 720, belong to the Cellular Component: ‘cytoplasmic part’ whereas 269 belong to the ‘nuclear part’. With Molecular Function ‘binding’ is substantially represented with 683 proteins involved, specified as ‘protein binding’ (420), ‘ion binding’ (369), ‘organic cyclic compound binding’ (325), ‘heterocyclic compound binding’ (324), ‘small molecule binding’ (224), and ‘anion binding’ (220). Additionally, several proteins are characterized with ‘catalytic activity’ (469). Proteins enriched in the Reactome Pathways were ‘metabolism’ (206), ‘metabolism of proteins ‘(159), ‘immune system’ (133), ‘post-translational protein modification’ (102), and ‘innate immune system’ (94). Thus, proteins found to be perturbed in HCT 116 were mainly cytoplasmic proteins with various binding functions, some having catalytic activity, that participate in protein metabolism and the immune system.

## 3. Discussion

A number of studies have been published on diagnostic/prognostic/predictive markers including hypothesis-generating proteomic analysis of CRC performed with patient samples, animal models and cell lines as reviewed by de Wit et al. [29]. In the present study, the sample material included the total protein content from cellular models established from a primary adenocarcinoma of the colon, SW480, a colonic carcinoma, HCT 116, and a normal derived colon mucosa cell line, NCM460. The two colorectal cancer cell lines show a number of differences [16]. SW480 from a Dukes’ B stage tumor shows intermediate growth while HCT 116 from a Dukes’ D stage tumor is fast growing. SW480 shows microsatellite stability while HCT 116 possesses microsatellite instability. SW480 and HCT 116 have different CpG island methylator phenotypes and different chromosomal instability status. Additionally, they show a number of differences in mutations of KRAS (G12V in SW480, G13D in HCT 116), PIK3CA (wt in SW480, H1047R in HCT 116) and TP53 (R273H and P309S in SW480, wt in HCT 116) [16]. In spite of these molecular differences as well as differences in specific proteins that are differentially expressed, the bioinformatic analyses showed a number of similarities with respect to enriched pathways among the two cell lines. 2D-PAGE and LFQ LC–MS/MS-based proteomic analyses have not previously been published with these cellular systems. The biological variation within a cell line is minimal as compared to patient samples. However, results from model systems show some differences in expression levels of proteins and must be further verified in patient samples to establish the clinical impact.

### 3.1. Model Systems Versus Patient Tissue

In the present analysis, we identified some differentially expressed proteins in one of the cell models that show similar expression patterns in the patient tissue (reticulocalbin (SW480), calumenin (SW480), S100A6 (SW480)). Other proteins showed a more complex pattern (protein SET, endoplasmin). We were unable to establish the expression pattern in the patient tissue for S100A4 and retinol binding protein I. The latter was highly upregulated in both cell lines, SW480 and HCT 116. The antibodies did not work on tissue, presumably because the protein composition may be different from the cellular model systems. Patient tumor tissue may also be heterogeneous and show varying degrees of central necrosis. Further a tumor may consist of different clones, which may have different protein composition. Therefore, we analyzed central as well as peripheral parts of the tumor. Thus, conclusions from the model systems cannot always be transferred to the clinical setting.

### 3.2. Proteomics Methodology

We have used 2D-PAGE where quantification is performed directly on intact proteins (top-down proteomics) as well as LFQ LC–MS/MS where quantification is performed on tryptic generated peptides (bottom-up proteomics). Proteoforms [30] of the proteins can be separated by 2D-PAGE if they vary in pI and/or molecular mass, which may include different posttranslational modifications and fragmentations. Since bottom-up proteomics is performed on peptides from digested proteins this technique does not give detailed information about proteoforms of the proteins. A major advantage of the latter technique is that it can be performed on formalin-fixed paraffin-embedded (FFPE) CRC patient tissue [31]. FFPE in combination with 2D-PAGE is still insufficient [32]. The use of frozen tumor tissue is applicable with 2D-PAGE.

### 3.3. Comparisons of 2-Dimensional Polyacrylamide Gel Electrophoresis (2D-PAGE) with 1D Western Blotting

**Figure 6:**
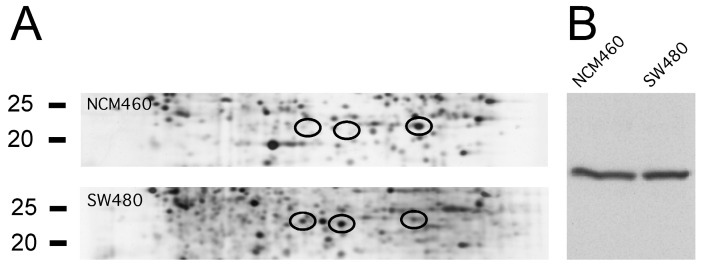
Expression of triosephosphate isomerase. (**A**) In 2-dimensional polyacrylamide gel electrophoresis (2D-PAGE), the protein migrates as at least 3 proteoforms (indicated with black circles) with varying isoelectric points but similar molecular masses that each vary in concentration. (**B**) When analyzed by 1D western blotting the proteoforms co-migrate as one band where the total concentration is similar in the two cell lines.

When 2D-PAGE is compared with 1D western blotting, there are some issues to be considered. In order to obtain consistent results, antibody specificity is crucial. The specificity may depend on the protein composition in the system used, as the combination of proteins may be different from the cell lines to tissue samples. Here, we report results from the antibodies that worked either in the cell lines and/or in the tissue. Another problem is that proteins may consist of several proteoforms that may migrate to different isoelectric points with similar molecular masses using 2D-PAGE. Differential expression of each of these single isoelectric variants may be detected by proteomic analysis using 2D-PAGE while this may not be apparent when all isoelectric variants are analyzed together as a single band using 1D western blotting. As an example, triosephosphate isomerase migrates as at least three proteoforms that vary in isoelectric points but with similar molecular masses that are expressed to varying degrees in NCM460 and SW480 (Figure 6A). However, the 1D western blot analysis revealed band intensities that were quite similar in the two cell lines (Figure 6B).

### 3.4. Differentially Expressed Proteins

#### 3.4.1. CREC Proteins

We found two of the three CREC proteins analyzed, reticulocalbin and calumenin, to be increased in the tumor cell line SW480, and both of them were also expressed to a higher degree in tumor biopsies (peripheral and central part) both compared to the non-involved part of the colon. They were not changed in the HCT 116 cell line. ERC-55, another CREC protein was expressed at similar levels in the cell model systems of normal derived cells, NCM460, and cancer cells, SW480 and HCT 116, and was not further examined. In the cellular model, the molecular masses of calumenin vary between the two cell lines (SW480 and NCM460) and regarding reticulocalbin more bands are observed in the tissue biopsies. Both of these expression patterns may be due to glycosylations as previously shown for CREC proteins [33,34,35]. Thus, the differential expressions and differences in putative glycosylation states of reticulocalbin and calumenin imply some role of these proteins in CRC pathogenesis.

#### 3.4.2. S100 Proteins

The S100 family consists of more than twenty members with a high degree of sequence and structural similarities. So far, almost all of the S100 family members are involved in cancer with different expression patterns. In SW480, S100A4 was downregulated while S100A6 was upregulated. S100A6 was upregulated in the central part of the tumor compared to the non-involved part of the bowel. This pattern is consistent with previous studies [36,37,38,39]. The interpretation of high S100A4 expression is not unequivocal. Some studies have found a positive [40] where other studies show a negative [41,42] impact of high S100A6 expression in regard to CRC. The divergent results of S100A4 expression may be based on not taking the cellular localization into consideration. Previously, it has been shown that nuclear S100A4 from adenocarcinomas of the colon or rectum is a negative predictor of disease-free and overall survival whereas cytoplasmic S100A4 was not associated with patient outcome [43]. In the HCT 116 cell line we only detected S100A10 with LFQ LC–MS/MS and it was unchanged.

#### 3.4.3. Retinol-Binding Protein I

Retinol-binding protein I (RBP) was highly upregulated in SW480 both in the 2D-PAGE proteomic analysis, with western blotting of the cell lines and also in the HCT 116 cell line by LFQ LC–MS/MS. However, no specific antibody reaction was obtained on the patient tissue. Previous studies have shown that low level of RBP is correlated to an increased risk of tumor recurrence [44]. Furthermore, Ali et al. have shown that upregulation of RBP might have a role in TGF-β/Smad4 signaling resulting in cancer progression [45]. Thus, the expression pattern of RBP is not fully established. Still, this protein may be an important player in progression and recurrence in CRC.

#### 3.4.4. Protein SET

Protein SET was differentially expressed in the SW480 cellular model although not in the HCT 116 model. Western blotting of SW480 showed a two-band pattern, likely identifying the α- and β-isoforms (TAF-1α (290 amino acids)), TAF-1β (277 amino acids)). The two isoforms are 98.7% identical and differs only in the N-terminal 1-47 amino acids. The top band in SW480 showed significant downregulation. A complex pattern was found in CRC tissue with several bands. Quantification of all bands together showed a significant upregulation of protein SET in the peripheral and central part of the tumor compared to the non-involved part of the colon. Previously, protein SET was found upregulated in HCT 116 regarding a putative effect in the COX-2 inhibitor celecoxib [46]. The specific role of protein SET in pathogenesis of CRC awaits further investigations.

#### 3.4.5. Endoplasmin

Endoplasmin was identified in two spots in the 2D-PAGE proteomic analysis of SW480. Spot 1801 (Figure 2, approximately 80 kDa) is downregulated in SW480, and spot 0607 (Figure 2, approximately 55 kDa) is upregulated. Both spots possess lower molecular masses than the deduced mass of endoplasmin (92.4 kDa). Western blots showed a main band at approximately 80 kDa, most likely corresponding to spot 1801, that was downregulated in the SW480 cancer cell line confirming the 2D-PAGE analysis. No pronounced bands with lower molecular masses were observed in the SW480 cellular model. In the HCT 116 cellular system endoplasmin showed no changes. CRC patient tissue showed a major band with a molecular mass of approximately 64 kDa, also lower than the deduced mass, with a slight but significant up-regulation in the peripheral part of the tumor compared to the non-involved colon. Additional bands with even lower molecular masses (approximately 40 kDa and approximately 25 kDa) were also detected in the tumor tissue (not shown). Previously, two studies have shown endoplasmin expression to be correlated with CRC [47]. Thus, our results together with these studies infer that the expression of endoplasmin is complex and may correlate with CRC.

### 3.5. Conclusions

The CREC proteins reticulocalbin and calumenin seem to have a role in CRC carcinogenesis whereas ERC-55, another family member, shows no differential regulation indicating a specific role of reticulocalbin and calumenin and not the CREC family as such. Both reticulocalbin and calumenin were found with high expression in the tumor compared to the healthy part of colon and must therefore be seen as potential markers of CRC. Furthermore, in this study, more putative markers have been identified including the S100 protein S100A4 and S100A6, RBP, protein SET, and endoplasmin which are observed to be differentially regulated either in one of the cellular model systems and/or in CRC patient tissue. Further studies must be performed to investigate the biological roles of the various identified putative markers of CRC aiming at elucidating the clinical relevance of these new potential disease makers.

## 4. Materials and Methods

### 4.1. Cell Lines

SW480 (ATCC^®^ CCL-228™), a cell line established from a primary adenocarcinoma of the colon (Dukes’ type B) and HCT 116 (ATCC^®^ CCL-247™), a cell line established from a male adult with colorectal carcinoma were obtained from ATCC. NCM460, a cell line derived from normal colon mucosa was obtained from INCELL Corporation LLC (San Antonio, TX, USA) through a licensing agreement. The cell lines were cultured in M3:BaseA^TM^ (M310A, INCELL Corporation LCC, San Antonio, TX, USA) enriched with 10% fetal bovine serum (FBS) Gibco^TM^ 10270-098 (Thermo Fisher Scientific, Waltham, MA, USA) to make M3:10A.

Confluent cells were washed three times in PBS-buffer without Ca^2+^ and Mg^2+^ and scraped off. Pellets were further washed with PBS-buffer and kept at −80 °C until use. Cell pellets were re-suspended in SDS sample buffer (LC2676, Invitrogen) for 1D western blotting and in 2D lysis buffer (9M Urea, 2% (vol/vol) Triton X-100, 2% (wt/vol) DTT, 2% (vol/vol) IPG-buffer (GE Healthcare, Buckinhamshire, UK)) for 2D-PAGE analysis and in lysis buffer (5% (wt/vol) SDS, 50 mM TEAB, pH 7.55) for LFQ LC–MS/MS analysis. Protein concentration was determined using the Non-Interfering Protein Assay (488250, Calbiochem^®^, Merck KGaA, Darmstadt, Germany) or by infrared spectrometry (Direct Detect Spectrometer, Merck KGaA, Darmstadt, Germany) [48].

### 4.2. Tissue Biopsies from Patients with CRC

All subjects gave their informed consent for inclusion before they participated in the study. The study was conducted in accordance with the Declaration of Helsinki, and the protocol was approved by The North Denmark Region Committee on Health Research Ethics (Project identification No. 2-16-4-0001-03) and the Danish data agency. This project was further approved (18 December 2017) by the National Committee on Health Research Ethics (Project identification No. 60819). Biopsies were obtained from ten patients with CRC, Table 1. From each patient 3 samples were collected, one from the central part, one from the peripheral part of the tumor, as well as one from the resection margin furthest from the tumor. The tissue samples were collected from the tumor and bowel just after the specimen was removed from the abdomen, rinsed in cold saline, frozen in liquid nitrogen, and stored at −80 °C. For protein extraction, initially, the tissue was rinsed with PBS-buffer if presence of excess blood was observed, then solubilized in IP 3-10 NL lysis buffer (9M Urea, 2% (vol/vol) Triton X-100, 2% (wt/vol) DTT, 2% (vol/vol) IPG-buffer (GE Healthcare)) using a homogenizer. Protein concentration was determined using the Non-Interfering Protein Assay (488250, Calbiochem^®^, Merck KGaA, Darmstadt, Germany).

### 4.3. Two-Dimensional Polyacrylamide Gel Electrophoresis (2D-PAGE)

2D-PAGE was done essentially as previously described [49]. Six replicates of each cell line were harvested and from each sample 75 µg of total protein was loaded on the gels. In short, first dimension isoelectric focusing (IEF) was performed using pH 3–10 nonlinear 18 cm strips (GE Healthcare). Separation in the second dimension was performed using polyacrylamide gels (12% T, 3% C). Gels were then silver stained and scanned on a GS-710 Imaging Densitometer (Bio-Rad, Hercules, CA, USA) using Quantity-One. The TIFF files were then imported into PDQuest software (BioRad, Hercules, CA, USA). Protein spots were initially automatically defined and quantified and then manually investigated for proper alignment between the gels. Differentially expressed spots were defined as spots that were 2-fold or more differentially expressed with a significance level at *p* < 0.05 (Mann-Whitney U-test).

### 4.4. Liquid Chromatography–Tandem Mass Spectrometry (LC–MS/MS) for Protein Identification

Differentially expressed spots were excised from either the rehydrated silver stained gels or for a few spots from Coomassie blue stained gels. The excised spots were subjected to in-gel tryptic digestion and peptides were identified by liquid chromatography–tandem mass spectrometry (LC–MS/MS) with a Q-Tof Premier mass spectrometer (Waters, Milford, MA, USA). Proteins were identified by searching in the SwissProt protein database (various releases from 52.5–57.11) using the online version of the Mascot MS/MS Ions Search facility (Matrix Science, Ltd., London, UK) [50]. The parameters used for searching were doubly and triply charged ions with up to two missed cleavages, a peptide tolerance between 20 and 100 ppm or 1 Da, one variable modification, Carbamidomethyl-C (occasionally together with oxidation of Met) and a MS/MS tolerance of 0.02 or 0.05. Contaminating peptides such as keratins and trypsin were disregarded. Individual ions scores above approximately 34–37 indicated identity or extensive homology giving a less than 5% probability that the observed match was a random event.

### 4.5. Label-Free Quantification Liquid Chromatography–Tandem Mass Spectrometry (LFQ LC–MS/MS)

Proteomic comparison of cell lines NCM460 with HCT 116 were performed with LFQ LC–MS/MS using an LC-MS instrument platform from Thermo Fisher Scientific (Waltham, MA, USA). This was performed essentially as previously described [51] with slight modifications. Protein preparation and tryptic digestions from each cell line was performed using the S-Trap Micro Spin Column procedure (Protifi, Farmingdale, NY, USA) as described by the manufacturer. In short, from each of the cell lines six 20 µg protein preparations were alkylated with TCEP and digested with trypsin. Peptide concentration was measured by fluorescence as described [48]. Samples were dried by vacuum centrifugation and dissolved in 0.1% formic acid. Peptides were separated by nano liquid chromatography, Ultimate 3000 (Thermo Fisher Scientific, Waltham, MA, USA). Peptides were trapped with a µ-Precolumn (300 µm × 5 mm, C18 PepMap100, 5 µm, 100 Å (Thermo Fisher Scientific, Waltham, MA, USA) and separated on an analytical column (EASY-Spray Column, 500 mm × 75 µm, PepMap RSCL, C18, 2 mm, 100 Å, Thermo Scientific) coupled to an Orbitrap Fusion Tribrid mass spectrometer (Thermo Scientific). Peptides were eluted with a flow of 300 nL/min. using a gradient by mixing buffer A (99.9% (vol/vol) water, 0.1% (vol/vol) formic acid) with buffer B (99.9% (vol/vol) acetonitrile, 0.1% (vol/vol) formic acid). One µg of peptide was injected from each preparation in duplicate. The universal method setting was used with full Orbitrap scans (m/z 375–1500) at a resolution of 120,000 with automatic gain control (AGC) target of 4 × 10^5^ and a maximum injection time of 50 ms. Most intense precursors were selected with an intensity threshold of 5 × 10^3^. Charge states 2–7 were included. In the linear ion trap MS2 scans were performed at rapid scan rate with collision-induced dissociation energy at 35%, an AGC target of 2 × 10^3^ and a maximum injection time of 300 ms. Precursor ions were isolated using the quadrupole set with an isolation window of 1.6 m/z. Cycle time was 3 s and dynamic exclusion was set to 60 s. Raw data files were used to search the reviewed human database from Uniprot downloaded on the 9th of February 2020 and using MaxQuant version 1.6.6.0 for LFQ analysis [52]. Carbamidomethyl (C) was used as fixed modification. False discovery rate for PSM, protein and identification by site was each set at 1%. The LFQ minimum ratio count was set to 1. MS/MS was required for LFQ comparisons. Unique and razor peptides, unmodified and modified with oxidation (M) or acetyl (protein N-terminal) were used for protein quantification. Contaminant sequences were included in the search and revert sequences were used for decoy search. The generated results file was further analyzed with Perseus version 1.6.6.0 [53]. Proteins identified in all samples in both groups were included together with proteins identified in all samples in one group but were below detection limit in all samples in the other group, since these are of high interest as putative biomarkers. Differentially expressed proteins were defined as proteins that were 2-fold or more differentially expressed with a significance level at *p* < 0.05 (*t*-test).

### 4.6. Western Blotting

Western blotting was done essentially as previously described [49]. In short, 30 µg of total protein from cell lines or 10 µg of total protein from tissue was loaded in each lane, separated by electrophoresis and blotted onto nitrocellulose Hybond-C Extra membranes. We used the total protein level for normalization of the blots and abstained from using household proteins. This was based on our previous experience that GAPDH and actin are differentially expressed in lymphoma tumor biopsies [49]. Furthermore, Hu and coworkers [54] have shown that seven commonly used household proteins change expression level in CRC in particular. Recently, Xu and coworkers [55] showed that 17 out of 21 classical reference genes were upregulated in CRC at transcript level compared to normal colonic epithelial tissue. Even among 42 novel potential reference genes some of these transcripts changed expression level in a subset of the tumors. Thus, the ideal reference gene or protein is still missing and we found it more reliable to normalize to the total protein as recently suggested [54]. All primary antibodies were incubated overnight at 4°C or for one hour at room temperature. The blots were then incubated with horseradish peroxidase (HRP)-conjugated secondary antibodies and developed using the enhanced chemiluminescence system. Digital images were taken using Fujifilm LAS-4000 or LAS-4010 and the corresponding ImageQuant LAS 4000 software (Fuji, Tokyo, Japan). The images were analyzed by ImageQuant TL, version 7.0. Pixel intensity for each band was measured and normalized to the background density. The intensities were quantified and Mann-Whitney U-test for the cell line studies and Wilcoxon signed rank test for the analyses of patient tissue were performed to identify significant differences. In the cell line blots, the mean value of NCM460 pixel intensity was set to 1 as a reference. All intensities were calculated relative to this reference. In the blots with patient tissue samples, the pixel intensity from the non-involved part of the colon (N) of each patient was set to 1 and pixel intensity from both the peripheral (P) and central (C) part of the tumor was calculated from this reference. All values were log_2_-transformed prior to visualization by dot plots in Figure 1 and Figure 4.

Primary antibodies: anti-reticulocalbin, anti-calumenin and anti-ERC-55 are all affinity-purified polyclonal antibodies generated and characterized in our laboratory [34,56,57]. The other antibodies were purchased from commercial suppliers: anti-RBP (ab31106, Abcam PLC, Cambridge, UK), anti-SET (NBP1-33713, Novus Biologicals, LLC, Centennial, CO, USA), anti-S100A4 (HPA007973, Atlas Antibodies AB, Bromma, Sweden), anti-S100A6 (ab11181, Abcam) and anti-endoplasmin (HPA008424, Atlas Antibodies). Secondary HRP-conjugated antibodies: rabbit anti-mouse PO260 HRP, swine anti-rabbit PO217 HRP and rabbit anti-goat PO449 HRP were purchased from Dako Denmark A/S (Glostrup, Denmark).

### 4.7. Bioinformatics

All MS identified proteins from 2D-PAGE analysis with two or more peptides (Table 2) together with S100A6, calumenin, and reticulocalbin were uploaded to STRING v11.0 (https://string-db.org/) [28] using the Uniprot entry names. For the bioinformatic analysis of proteins identified with LFQ LC–MS/MS all 901 proteins listed in supplementary Table S1 were uploaded and STRING recognized 899. In the STRING analysis we focused on the Gene Ontology aspects, Molecular Function and Cellular Component as well as pathways identified from the Reactome pathway database [58].

## Figures and Tables

**Table 1:** Patient characteristics.

Patient no.	Sex	Age by the Time of Diagnosis	Localization	T	N	M	Rt-Type	Recurrence	Death Due to CRC
3	M	63	0	4	2	1	0	NA	Y
13	M	73	0	3	1	1	0	NA	Y
17	M	58	0	4	2	0	0	Y	Y
20	F	75	1	2	0	0	2	N	-
25	M	65	0	3	0	0	0	Y	-
40	M	69	0	2	0	0	0	N	-
46	M	76	0	3	0	0	0	Y	-
48	F	80	0	3	0	1	0	NA	Y
78	F	54	0	4	1	1	0	NA	Y
97	M	79	0	3	0	0	0	N	-

Characterization of patients included in the project. Sex: Female(F)/ Male(M), Age by the time of diagnosis: in years, Localization: of the tumor 0 = colon 1 = rectum, TNM Clinical Classification T = primary tumor 1–4 N = Regional Lymph Nodes 0–2 M = Distant Metastasis 0–2, Rt-type = Radiotherapy 0–2 (0= no radiotherapy 1 = long-course 52Gy/26 fractions 2 = Short-course 25Gy/5 fractions) Recurrence: Y = yes N = no NA = not appropriate, Death due to CRC: Y = yes N= no.

**Table 2:** Identification of differentially expressed proteins in SW480 versus NCM460 cells.

Ratio SW480/NCM460	SSP	Identification	Peptides	Mascot Score	Mr (Da)	SwissProt Name
0.15	9002	Transgelin-2	2	48	22377	TAGL2_HUMAN
0.15	6204	Delta(3,5)-Delta(2,4)-dienoyl-CoA isomerase, mitochondrial	1	49	35793	ECH1_HUMAN
0.17	2804	Transitional endoplasmic reticulum ATPase	6	269	89958	TERA_HUMAN
		Src substrate protein p85	2	80	63462	SRC8_HUMAN
0.20	4001	Protein S100-A4	2	125	11721	S10A4_HUMAN
0.24	1801	Endoplasmin fragment	9	405	92696	ENPL_HUMAN
0.26	4109	Endoplasmic reticulum protein ERp29	1	75	28975	ERP29_HUMAN
0.27	2902	Hypoxia up-regulated protein 1	11	401	111494	HYOU1_HUMAN
0.28	3601	FK506-binding protein 4	6	366	52057	FKBP4_HUMAN
0.28	3705	Heat shock cognate 71 kDa protein	3	170	70854	HSP7C_HUMAN
		Lamin-B2	1	110	67647	MMNB2_HUMAN
		Stress-70 protein mitochondrial	3	87	73635	GRP75_HUMAN
0.29	8608	Adenylyl cyclase-associated protein 1	1	46	51823	CAP1_HUMAN
0.33	8701	Heterogeneous nuclear ribonucleoprotein L	3	70	60719	HNRPL_HUMAN
0.35	1501	Tubulin beta	3	229	ca. 50000	TBB2C_HUMAN **
0.38	1002	Thioredoxin	1	52	12015	THIO_HUMAN
0.39	9003	Growth factor receptor-bound protein 2, fragment	3	75	25190	GRB2_HUMAN
0.42	9207	Voltage-dependent anion-selective channel protein 1	1	107	30722	VDAC1_HUMAN
0.42	1603	Tubulin alpha	4	195	ca. 50000	TBA1B_HUMAN **
0.43	5303	60S acidic ribosomal protein P0-like	1	56	34343	RLA0L_HUMAN
0.45	8206	Annexin A2	1	53	38580	ANXA2_HUMAN
		Putative annexin A2-like protein			38635	AXA2L_HUMAN
0.46	8204	Annexin A2	2	120	38580	ANXA2_HUMAN
		Putative annexin A2-like protein			38635	AXA2L_HUMAN
0.46	6603	T-complex protein 1 subunit beta	6	418	57452	TCPB_HUMAN
0.46	6202	Proteasome subunit alpha type-1	2	70	29537	PSA1_HUMAN
0.48	7403	Elongation factor Tu, mitochondrial	1	42	49510	EFTU_HUMAN
2.07	3206	Tubulin alpha, fragment	1	64	ca. 50000	TBA1B_HUMAN **
2.07	0401	Putative heat chock protein HSP 90-beta-3, fragment	1	42	68282	H90B3_HUMAN
2.11	3104	Prohibitin	4	131	29786	PHB_HUMAN
2.14	0301	Nucleophosmin	1	56	32555	NPM_HUMAN
2.16	0304	Protein SET	1	60	33469	SET_HUMAN
2.26	0607	Endoplasmin, fragment	4	170	92411	ENPL_HUMAN
2.76	0101	Acidic leucine-rich nuclear phosphoprotein 32 family member A	1	89	28568	AN32A_HUMAN
3.17	5003	Vesicle-associated membrane protein-associated protein B/C, fragment	4*	156	27211	VAPB_HUMAN
3.28	3202	Tubulin beta, fragment	1	65	ca. 50000	TBB5_HUMAN **
6.08	3002	Cellular retinoic acid-binding protein 2	7*	401	15683	RABP2_HUMAN
6.65	4004	Cofilin-1, fragment	3	158	18507	COF1_HUMAN
8.58	5105	Triosephosphate isomerase	4	274	26698	TPIS_HUMAN
17.94	0008	Nucleophosmin, fragment	1	77	32555	NPM_HUMAN
18.45	3208	ATP-dependent DNA helicase 2 subunit 2, fragment	1	88	82652	XRCC5_HUMAN
99.68	0009	Nucleophosmin, fragment	1	48	32555	NPM_HUMAN
378.64	1007	Retinol-binding protein I, cellular	1	69	15840	RET1_HUMAN
			3 *	182		
			4 *	147		

* Identified from Coomassie stained gels. The rest is identified from silver stained gels. ** Several variants.

## Supplementary Materials

Supplementary materials can be found at https://www.mdpi.com/1422-0067/21/10/3466/s1. Table S1: The differentially expressed proteins. Figure S1: Bioinformatic analysis of differentially expressed proteins in HCT 116 cells versus NCM460 cells obtained by LFQ LC-MS/MS.

## Abbreviations

1D	1-dimensional
2D-PAGE	2-dimensional polyacrylamide gel electrophoresis
CEA	Carcinoembryonic antigen
CRC	Colorectal cancer
CREC	Acronym for *C*ab45, *r*eticulocalbin, *E*RC-55, *c*alumenin
LC-MS/MS	Liquid chromatography - tandem mass spectrometry
LFQ	Label-free quantification
wt	Wild-type
