# Supplementary material for: Proteomic Characterization of Colorectal Cancer Cells versus Normal-Derived Colon Mucosa Cells: Approaching Identification of Novel Diagnostic Protein Biomarkers in Colorectal Cancer
Source: Int J Mol Sci. 2020 May 14;21(10):3466. doi: 10.3390/ijms21103466 (PMC7278953; doi:10.3390/ijms21103466)
Supplement: Supplementary file 1 [file ijms-21-03466-s001.zip › Legend to Supplementary Figure 1.pdf]

Legend

Supplementary Figure 1. Bioinformatic analysis of differentially expressed proteins in HCT 116 cells versus NCM460 cells obtained by LFQ LC-MS/MS. STRING v11.0 was used to analyze the differentially expressed 901 genes listed in Supplementary Table 1 (899 were recognized by STRING). The proteins have significantly more interactions (7610 edges) than the expected 5050 ($p<1x10^{-16}$). The analysis indicated that the proteins are at least partially biologically related. Details are given in the text.
