# Supplementary figures and images for: Proteomic Characterization of Colorectal Cancer Cells versus Normal-Derived Colon Mucosa Cells: Approaching Identification of Novel Diagnostic Protein Biomarkers in Colorectal Cancer
Source: Int J Mol Sci. 2020 May 14;21(10):3466. doi: 10.3390/ijms21103466 (PMC7278953; doi:10.3390/ijms21103466)

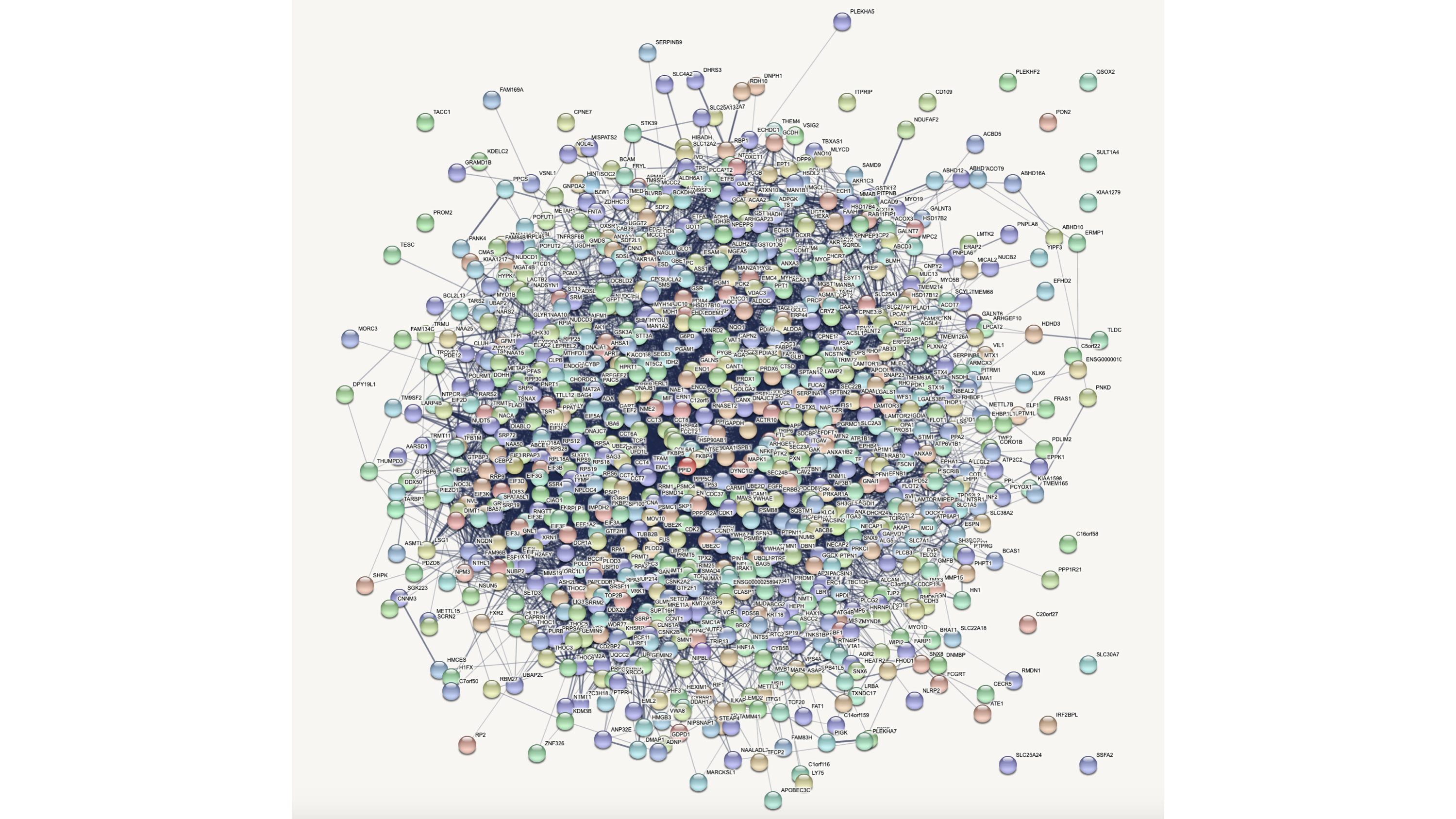

Supplement: Supplementary file 1 [file ijms-21-03466-s001.zip › Supplementary Figure 1.tiff]
